# A New Approach to Recording Rumination Behavior in Dairy Cows

**DOI:** 10.3390/s24175521

**Published:** 2024-08-26

**Authors:** Gundula Hoffmann, Saskia Strutzke, Daniel Fiske, Julia Heinicke, Roman Mylostyvyi

**Affiliations:** 1 Department Sensors and Modelling, Leibniz Institute for Agricultural Engineering and Bioeconomy (ATB), 14469 Potsdam, Germany; 2 Gouna GmbH, 14621 Schönwalde-Glien, Germany; 3 Ministry of Agriculture, Environment and Climate Protection of the Federal State of Brandenburg, 14467 Potsdam, Germany; 4 Faculty of Biotechnology, Dnipro State Agrarian and Economic University, 49600 Dnipro, Ukraine; mylostyvyi.r.v@dsau.dp.ua

**Keywords:** dairy cows, sensor, rumination, regurgitation, accelerometer

## Abstract

Rumination behavior in cattle can provide valuable information for monitoring health status and animal welfare, but continuous monitoring is essential to detect changes in rumination behavior. In a previous study validating the use of a respiration rate sensor equipped with a triaxial accelerometer, the regurgitation process was also clearly visible in the pressure and accelerometer data. The aim of the present study, therefore, was to measure the individual lengths of rumination cycles and to validate whether the sensor data showed the same number of regurgitations as those counted visually (video or direct observation). For this purpose, 19 Holstein Friesian cows equipped with a respiration rate sensor were observed for two years, with a focus on rumination behavior. The results showed a mean duration of 59.27 ± 9.01 s (mean ± SD) per rumination cycle and good agreement (sensitivity: 99.1–100%, specificity: 87.8–95%) between the two methods (sensor and visual observations). However, the frequency of data streaming (continuously or every 30 s) from the sensor to the data storage system strongly influenced the classification performance. In the future, an algorithm and a data cache will be integrated into the sensor to provide rumination time as an additional output.

## 1. Introduction

The measurement of rumination time (RT) in cattle is an important diagnostic method used in practice and research. The chewing that occurs during eating and ruminating is essential for digestion and the passage of feed through the gastrointestinal tract of the animal. It reduces the particle size of feed and increases saliva secretion, which helps to buffer the rumen environment and optimizes fiber digestion [1]. Rumination is spread out over several periods (up to 14) throughout the day. The cumulative daily time of rumination varies according to season, species, and diet and lasts 276–624 min/day in adult cows. One rumination cycle is composed of four phases (regurgitation, remastication, resalivation, and redeglutition) and takes approximately 45 s to one minute to complete. The cycle begins with the regurgitation of a food mass bolus and is accomplished by taking a breath (inspiration) with a closed glottis [2,3,4]. It is known that rumination is voluntarily controlled and that the cow is able to interrupt or stop rumination when disturbed [5]. Rumination also exhibits a diurnal rhythm, varying according to year, parity, the temperature–humidity index, daily milking and feeding times as well as days in milk [6].

Stress, caused by (subclinical) diseases [7,8,9,10] and summer heat [11,12,13,14] as well as physiological processes, such as estrus [15,16] and feed intake [17,18], can have an effect on RT. Rumination is also reduced on the day of calving in both healthy and sick cows, regardless of season [19]. Furthermore, RT can provide indications of disease processes during the transition period [20] and in the overall peripartum phase [21,22].

Continuous monitoring of the animal herd is essential for the timely detection of the time of estrus, reduced welfare, and health conditions. Altogether, RT monitoring offers opportunities for the early detection of individual health conditions and the optimization of reproduction in dairy cows [23]. Therefore, the importance of RT in the monitoring of health and well-being has led to the development of a variety of systems marketed to allow continuous monitoring of RT. In general, there are four indirect and automatic methods for monitoring RT. One widespread method in research is a pressure sensor attached to a noseband for measuring chewing behavior [24,25,26,27]. Another common option is to measure RT via triaxial acceleration sensors, for example, on the ear [28], collar [29], or halter [30] or in the rumen [31]. The third option is to use acoustic sensors such as a microphone [32,33,34,35] attached to the animal’s collar, and the fourth option is to monitor RT by using cameras [36,37]. Recent studies have also suggested that machine learning classification of images will become one of the important technological methods used for the intelligent housing and breeding of dairy cows in the future and have tested its use in rumination recording [38].

The objective of this study was to determine whether acceleration data from a sensor placed in the nose can be used to calculate the RT of an individual cow. The nasal sensor was originally developed to measure the respiratory rate in cows to replace the current standard of visual counting [39]. When analyzing the respiratory rate recordings in the original study at that time, it was observed that each time a bolus was regurgitated, there was a characteristic pattern in the pressure data as well as in the accelerometer data. These patterns were analyzed in more detail in the present study by comparing visually observed regurgitations (the gold standard) and sensor-detected rumination events.

## 2. Materials and Methods

### 2.1. Animals and Housing

This study was conducted at the Educational and Experimental Center for Animal Breeding and Husbandry (LVAT, Groß Kreutz, Germany) during February 2018, January 2022, and June 2022. In total, 19 Holstein Friesian cows (6 and 13 cows in 2018 and 2022, respectively) were used in this study, and data were recorded on different days and times between 0800 h and 1800 h. The lactating dairy cows were housed in a free-stall barn equipped with 53 lying cubicles (straw–lime mixture as bedding), and the herd size varied between 52 and 55 cows. The cows were milked 2.4 ± 0.5 times per day (mean ± SD) with an average milk yield of 39.79 ± 7.21 kg and days in milk of 122 ± 54.9 on the first day of the trial. The animals differed in their stage and number of lactations (1st to 5th lactation) as well as in their age (2.09 to 6.61 years at the beginning of their trial entry) and reproductive status (8 non-pregnant and 11 pregnant cows). This ensured that the method could work under different animal-specific conditions. During the trial, the animals were able to move freely in the barn (i.e., their natural behavior was not restricted). The automatic milking system, fresh water, and a total mixed ration were always freely available. The total mixed ration consisted of maize and grass silage, feed straw (triticale), fodder rye, and alfalfa silage for roughage as well as corn maize and soybean meal, calcium salt, and summer barley grist for concentrated feed (further details can be found in [14]).

### 2.2. Sensor Data

**Figure 1:**
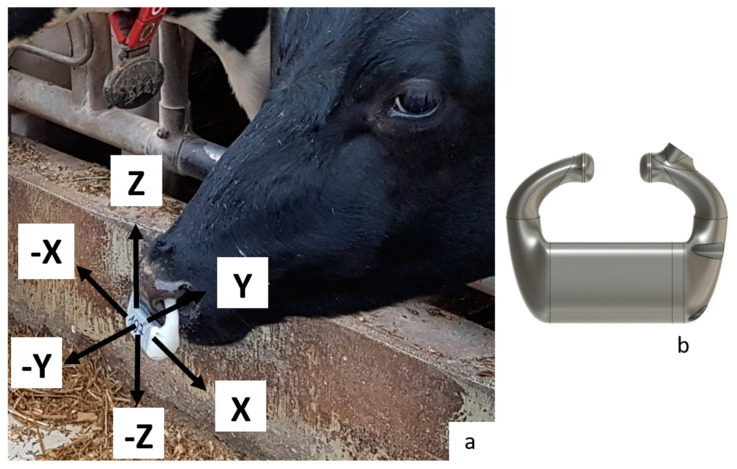
(**a**) Respiration rate sensor attached to the nose of a cow and orientation of the 3 axes of the inserted acceleration sensor (*x*-, *y*-, and *z*-axes; perpendicular to each other). (**b**) Drawing of the sensor.

The sensor-based data were recorded with a respiration rate sensor (for further details, see Strutzke et al. [39]) fixed at the head of the cows. An internal triaxial acceleration sensor (maximum value: 1 g, measured value for x: ±0.25 g) was included in every nose clip (weight: 72 g, width: 9.5 cm, height: 7.0 cm), and the pressure from nasal inhalation and exhalation was measured by a differential pressure sensor (see Strutzke et al. [39]). When implementing the pressure sensor and the acceleration sensor, the size, weight, and measuring range of the sensor components were decisive. On the one hand, the overall system had to be as light as possible for the cow’s nose, and, on the other hand, the measuring range had to be sensitive enough to be able to measure the pressure differences between breathing and the acceleration values of various nose movements. This sensor and a power bar were fixed via a halter in 2018, whereas in 2022, the power was supplied by a battery, and a halter was no longer needed (the sensor and battery were included in the nose clip fixed directly in the nose of the cow, Figure 1a,b). Data transmission always occurred wirelessly via a local network to a server, with the data streamed continuously (live observations) in 2018 and every 30 s in 2022. The battery life was prolonged to several weeks by reducing data transmission to once every 30 s.

### 2.3. Visual Observations

Observations of rumination behavior via video and direct observations were performed to validate the possible use of the sensor data for recording RT. The prerequisite for behavioral observations was that the cow was standing or lying calmly without external disturbances, e.g., interactions with other cows. However, videos (Samsung Galaxy Note 10.1, Seoul, South Korea) were recorded in 2018, and direct observations were performed in 2022. For video analysis in 2018, each of the six cows was filmed 3 to 4 times during rumination, and the length of the recordings was 9.8 ± 5.1 min (mean ± SD), resulting in a total of 187 min of video material. During the present study, trained personnel (veterinarians and agricultural scientists) performed the analysis of the videos and the direct observations. The suitability of human observations as the reference method for rumination observation was proven by Schirmann et al. [32] and Elischer et al. [35], with independent observers.

For the direct observations in the barn in 2022, the cows were chosen randomly by looking for ruminating cows with an attached respiration rate sensor in a standing or lying position. Therefore, 2 out of the 13 cows were observed at two times on different days. The observer chose a position near the head of the cow where the cow’s regurgitations were visible without disturbing the cow and noted the regurgitations until the cow stopped its rumination behavior. Regurgitation was defined as the moment when the cow interrupted chewing to bring up a bolus into the mouth to rechew. The time point of every regurgitation was written down (h:m:s) on a paper form and in an Excel file afterward. The duration of each observation period was between 5 and 56 min (mean: 00:24:52), with 15 observation periods and 375 min of observed rumination behavior collected in total.

As a negative control, six 10 min videos were recorded in 2018, and 6 cows were observed for 10 min each in 2022 while they were not ruminating, e.g., while they were dozing, feeding, standing, or lying without exhibiting rumination behavior. During these periods, we checked whether other behaviors could cause similar patterns in the sensor-based recordings. If an animal started to ruminate during this time, the video recording or direct observation was interrupted and started again with another sensor-wearing animal.

### 2.4. Data Processing

**Figure 2:**
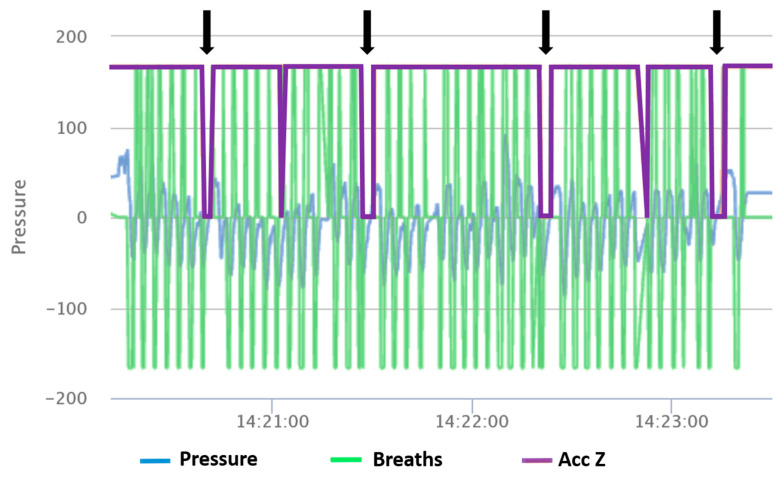
Sensor data: Pressure during inhalation and exhalation (blue), representation of single breaths (green), and regurgitation events (Acc Z, purple) recorded by the internal acceleration sensor (without units because of adjustments for graphical illustration). Four regurgitations are visible in the above figure (black arrows).

The associated sensor-based data from all observed time periods (regardless of the behavior shown) were saved, and visualizations of data were generated in Excel (MS Office 2016, Microsoft, Redmond, WA, USA). The data in the figures were all analyzed by one trained person who counted the characteristic regurgitation patterns, blinded to whether the data were from negative controls. The characteristic patterns were visible by a value (*y*-axis) near 0 of the z-orientation for at least 2–4 s, often followed by a longer exhalation or inhalation (wider pressure peak upward or downward, respectively) due to the pause in breathing (Figure 2).

Then, the time points (*x*-axis values) were recorded in an Excel file along with the time points of the visually observed regurgitation events. If the sensor data suggested regurgitation at the same time point (±5 s) as the visual observer, it was marked as a true positive observation. If no regurgitation was visible during the negative control, one minute was counted as one true negative observation.

### 2.5. Statistical Analysis

Statistical analysis was performed in JMP (16.1.0, SAS Institute, Cary, NC, USA). In the first step, the duration of the individual rumination cycles was analyzed by calculating the differences between the two following regurgitations for the visually recorded regurgitation times in 2022.

In the second step, the regurgitation detection performance by the sensor was measured using binary classification. Data from 2018 and 2022 were analyzed separately, as an additional analysis with corrected data (elimination of existing transmission errors that resulted in data gaps) was performed in 2022. The sensitivity (*Se*), specificity (*Sp*), positive predictive value (*PPV*), and negative predictive value (*NPV*) were used for the three situations (2018, 2022 raw data, 2022 corrected data) using the following equations:

(1) $$\mathrm{Se}\;[\%]=\frac{TP}{\left(TP+FN\right)}\times 100$$

(2) $$\mathrm{Sp}\;[\%]=\frac{TN}{\left(TN+FP\right)}\times 100$$

(3) $$\mathrm{PPV}\;[\%]=\frac{TP}{\left(TP+FP\right)}\times 100$$

(4) $$\mathrm{NPV}\;[\%]=\frac{TN}{\left(FN+TN\right)}\;\times \;100\;$$

where *TP* = number of true positive observations, *FN* = number of false negative observations, *TN* = number of true negative observations, and *FP* = number of false positive observations. 

The correction of the 2022 data was performed prior to testing classification performance by removing both the number of regurgitations from *FN* and the missing minutes from *TN* that could not be recorded by the sensor because of transmission errors.

## 3. Results

### 3.1. Duration of Rumination Cycles

**Figure 3:**
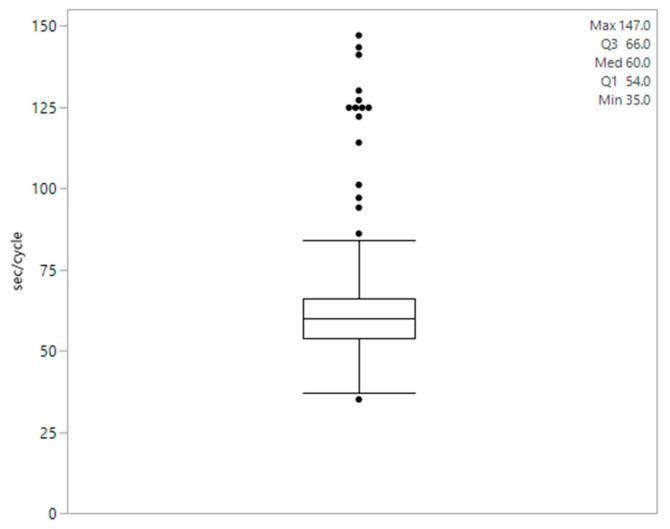
Distribution of the duration per rumination cycle in seconds (*n* = 13 cows).

Regarding the duration of each rumination cycle (*n* = 359), we found that the values did not follow a normal distribution. The median duration was 00:01:00 (h:m:s), with a minimum of 00:00:35 and a maximum of 00:02:27 per cycle. The distribution of the cycle lengths is shown in Figure 3. Recalculation of the duration of rumination cycles after deleting the outliers recorded in one cow with nonphysiological values led to a normal distribution and a mean duration of 59.27 ± 9.01 s per cycle.

### 3.2. Number of Regurgitations

A comparison of the trials in 2018 and 2022 and the observed regurgitations (visual or via sensor) during the rumination phases and during the negative control is shown in Table 1. There were gaps in the sensor-based data (pressure and accelerometer data), which resulted in missing regurgitation events (*n* = 149) and missing minutes in the negative control (*n* = 19). The reason for these gaps was the streaming of data every 30 s in 2022. During this frequency of data transmission, the sensor was not able to store the pressure values or accelerometer orientations, resulting in gaps.

### 3.3. Classification Performance

The comparison of sensor-based data with visually observed data (video or directly observed regurgitations) revealed different performances in the two years (Table 2). 

Regarding the streaming gaps, a correction was performed to exclude unclear sensor values, and *FN*s were corrected by removing regurgitations that could not be recorded by the sensor because of the streaming gaps (*n* = 149). Furthermore, *TN*s were reduced according to the number of minutes with streaming gaps in the sensor data (*n* = 19), and the performance was recalculated.

## 4. Discussion

Visual observations are still the gold standard for precise recordings of animal behavior, but they are time- and labor-intensive. Wearable sensors can be a more practical way to collect behavioral data over longer periods of time. Therefore, an increasing number of sensor systems [40,41] have been developed, but contactless camera techniques such as depth and video cameras [36,37] are often used to measure physiological and behavioral data in both research and practice. In practical conditions, farmers need easy-to-use early warning systems to reduce their workload and observation time. Steeneveld et al. [42] concluded that labor reduction is an important reason for investing in sensor systems for dairy cows.

Rumination behavior is a very important indicator for animal welfare, health, estrus, and calving detection [5,6,7,8,9,10,11,12,13,14,15,16,17,18,19]. A decrease in daily RT can be an early warning signal, but the chews per bolus, the number of regurgitations per day, and the duration per rumination cycle can also be analyzed to detect pathological conditions.

On closer inspection, we found a median duration of 60.0 s per rumination cycle (the period between two regurgitations). This is at the upper limit of the reference value of 45–60 s [4] and is slightly higher than the values of a German study reporting a mean duration of 57.31 ± 5.34 (mean ± SD) recorded with an electromyography-based sensor system [43]. A closer review of the values of individual cows revealed that one cow in the present study showed obviously atypical mean values of approximately 2 min per cycle (118.57 ± 23.09 s). Therefore, this cow was considered an outlier, and the removal of data from this cow and subsequent recalculation of all values resulted in a normal distribution and a mean duration of 59.27 ± 9.01 s per cycle in this study. This is in good agreement with the previously mentioned German study by Wobschall [43] and with Frandson et al. [5], who stated that one rumination cycle requires approximately 1 min, of which 3 to 4 s is used for both regurgitation and reswallowing.

The one cow with an atypical duration of rumination cycles highlights the importance of monitoring each individual animal on a farm. Here, a closer look and a consultation with a veterinarian can be helpful to identify the reason for this deviation. Possible reasons could be dental problems or pain during regurgitation (e.g., injuries of the esophagus) or a changed feed structure, if the prolonged rumination cycle was observed in more than one cow. The importance of individual measurements was also confirmed by Byskov et al. [18], who found that 48% of the total variation in RT is due to individual variation among cows. It should also be noted that cows are capable of voluntarily interrupting or stopping the rumination process, although it appears to be largely reflexive [5].

Therefore, a sensor that measures not only RT but also the duration of rumination cycles can be a helpful method for automatic health observation in dairy cows. In the present study, we tested such a sensor. It registered regurgitations that were further used to calculate both the RT per time interval (e.g., per day) and the duration of rumination cycles. The regurgitations visible in the sensor data were in good agreement (sensitivity: 99.1–100%, specificity: 87.8–95%) with visually observed regurgitations (on videos or directly) after the correction of sensor gaps due to data streaming settings. The comparison with sensor and vision systems of other studies is shown in Table 3.

Measurements of eating and rumination variables with a noseband pressure sensor in a barn also showed good agreement with data obtained via direct observation, with Spearman correlation coefficients ranging from 0.98 to 1.00 [26] and 0.91 to 0.96 [44]. This was confirmed by two other studies utilizing a pressure sensor in grazing cows. The concordance correlation coefficient (CCC = 0.94 and 0.96), which reflects the concordance between the sensor and visual observations, for the number of rumination chews shows a good correlation [27,45]. A similar system with a transducer to transform jaw movements into electrical signals overestimated RT (4.7% longer) compared with visual records, whereby rumination was more accurate than estimation of eating because the transducer was unable to distinguish between jaw movements caused by eating and grooming activities [25]. This highlights the advantage of the sensor used in the present study, where a typical pattern was found in the accelerometer data from the sensor attached to the nose during every regurgitation.

Other studies have also recommended the use of accelerometers to record rumination and other behaviors in dairy cows, describing them as the most commonly used motion sensors [46]. This is in agreement with a study about an ear-attached triaxial accelerometer sensor and the correlation (Pearson correlation coefficient and CCC values) of 0.93 between sensor-based and visually observed RT [28]. The ear seems to be a better position than a collar placed on the left side of a cow’s neck for a commercial triaxial accelerometer system. The collar-based sensor system produced lower correlations (Spearman: r = 0.83, CCC = 0.86) between visual observations and sensor-based data than the ear sensor for RT. However, the limits of agreement for rumination remained quite wide in that investigation (−15.80 min/h, 15.00 min/h) [29]. The most comparable methods with those used in the present study (the position of the respiration rate sensor in the nose) were described by Ding et al. [30]. In their study, three mini triaxial accelerometers were integrated into a tightly adjusted halter and placed over the nasolabial levator muscle, the right masseter muscle, and the left lower lip muscle; data were collected at 1 Hz and later compared with video observations. The developed behavior identification model recognized different jaw movement activities with a precision of 99%, and the best accuracy for recording feed intake was reached with sensors positioned over the nasolabial levator muscle. In comparison with the nose clip used in the present study, the halter [30] and the noseband pressure sensor [26,27,44,45] should be positioned tightly to the cow’s head without disturbing the cow. In contrast, the nose clip must be selected in the appropriate size for the nose and may be more easily misplaced.

**Table 3:** Comparison of the discussed methods used to analyze rumination behavior.

Kind of Sensor System	Statistical Method	Accuracy Value	Reference
Noseband pressure sensor	Spearman correlation coefficient (SCC)	0.98–1.00	Braun et al. [26]
Noseband pressure sensor	SCC	0.91–0.96	Zehner et al. [44]
Noseband pressure sensor	Concordance correlation coefficient (CCC)	0.94	Werner et al. [27]
Noseband pressure sensor	CCC	0.96	Li et al. [45]
Accelerometer sensor, ear-attached	Pearson correlation coefficient (PCC)	0.93	Bikker et al. [28]
	CCC	0.93	
Accelerometer sensor, collar-attached	SCC CCC	0.83 0.86	Leso et al. [29]
Accelerometer sensor, halter-attached	Precision	99%	Ding et al. [30]
Accelerometer sensor, reticulum bolus	F1-score	0.86	Hamilton et al. [47]
Accelerometer sensor, reticulum bolus	PCC	0.72	Capuzzello et al. [31]
Acoustic sensor, collar-attached	PCC	0.93	Schirmann et al. [32]
Acoustic sensor, collar-attached	PCC	0.65	Elischer et al. [35]
Acoustic sensor, halter-attached	F1-score	0.75	Chelotti et al. [33]
Video	Success rate	92.03%	Chen et al. [36]
Video and modified YOLOv8-pose model	Accuracy	94.4%	Li et al. [48]
Triaxial vision system	Sensitivity	0.97	Song et al. [37]
Accelerometer sensor combined with differential pressure sensor, nose clip	Sensitivity Specificity	99.1–100% 87.8–95%	Hoffmann et al. [present study]

Triaxial accelerometers were also used by Hamilton et al. [47] and Capuzzello et al. [31], who both placed them in the rumen of the cow. Hamilton et al. [47] analyzed the motion from an accelerometer within a rumen bolus using a linear Support Vector Machine model to identify rumination periods with an overall accuracy of 86.1%. Capuzzello et al. [31] also analyzed the proportion of time spent ruminating in rumen-fistulated cows, using a bolus placed in the reticulum. Furthermore, they equipped the cows with neck collars and used ultrasound and auscultation as traditional methods to determine reticulo-ruminal contractility. Bland–Altmann plots showed similar performance with small biases regarding the intercontraction intervals based on bolus, ultrasound, and auscultation data. However, the Pearson correlation coefficient for the time spent ruminating derived from the neck collar and bolus methods was 0.72 in that study.

Accelerometers seem to be best for measuring rumination behavior, and attachment to the head looks the most promising, which is an advantage of the triaxial accelerometer used in the present study. However, this nasal sensor is very specialized for detecting rumination behavior and respiration rate. For example, it cannot distinguish between the lying and standing positions of a cow, which was noted by Vanrell et al. [46] as a disadvantage of using accelerometers on a halter for activities that can take place during different postures.

Another option to measure RT is to use an acoustic sensor such as a microphone to register distinctive sounds produced during regurgitation and rumination. A commercially available electronic rumination monitoring system involving a neck collar fixed on the left side of the neck was validated by Schirmann et al. [32]. The comparison of the electronic system with visual observations showed a high correlation (Pearson correlation: r_p_ = 0.93), indicating that the electronic system is suitable for monitoring rumination behavior in dairy cows. In contrast, in another study, the correlation between microphone-based measurements of rumination from the neck and visual observations of rumination resulted in a lower correlation coefficient (r_p_ = 0.65). The authors noted that incorrectly placed collars (too high, low, loose, or tight) could explain that result. An awkward position at the neck may interfere with the recording of rumination sounds by the monitor [35]. Unfortunately, data are calculated and summarized only in minutes per 2 h interval by this system [32,35], which is a disadvantage compared with other systems such as the sensor validated in the present study.

Another study also used acoustic signals for the recognition and estimation of grazing and rumination behavior. Specifically, a microphone was placed on the forehead of the cows. F1-scores (computed as a general performance indicator) higher than 0.75 were achieved for behavior classification. Similar to our sensor system, they also aimed to implement the method in a microcontroller-based system to provide online data in real time [33]. On the other hand, Chen et al. [36] pointed out that the accuracy of monitoring rumination behavior with microphones is limited by a variety of physical factors, such as noise caused by collision, friction, and shaking on the sensors. They preferred a video-based analysis of RT in their own trial and subsequently extracted cow mouth motion from the video. The results showed an average monitoring success rate of 92.03%. In this study, only individual animals were manually filmed during rumination, and a certain position of the cow’s head (not overly turned or raised) was required. This also applies to the study of Li et al. [48], who proposed a non-contact video method for monitoring cattle rumination behavior, utilizing an improved YOLOv8-pose keypoint detection algorithm combined with multi-condition threshold peak detection to automatically identify chewing counts. The improved YOLOv8-pose achieved a mean average precision of 96% in keypoint detection. However, the model struggled to detect the keypoints when the cow turned its head away from the camera, failing to identify the cow’s head and its corresponding keypoints. For rumination behavior, the average error in chewing count was 5.6%, with a standard error of 2.23% and an accuracy of 94.4% [48]. In [37], a sensitivity of 0.97 was achieved using a different video-based approach with a triaxial vision system mounted above an automatic milking system. This system is noninvasive, and only a few systems per herd are required, but it can only measure the reticulo-ruminal motility at single time points without measuring RT or the duration of rumination cycles, in contrast to the approach used in the present study. Furthermore, the environment in a dairy farm is complex and image features are easily affected by the illumination or background. In addition, at different growth stages, cow behavior classification results of the same model are also quite different [49], and camera systems require regular cleaning from dust and dirt and need a good IT infrastructure.

Comparisons of different technologies are difficult and, as discussed above, different statistical approaches are often used to calculate the performance of different devices (Table 3). However, the values (Spearman or Pearson correlation coefficient, concordance correlation coefficient, or F1-score) were in the overall range of 0.65 to 1.00 regarding the relationship between technically recorded rumination behavior and visual observations. Most of the validated devices seem to provide accurate monitoring of rumination behavior in dairy cows. This is in agreement with a validation study that monitored feeding, rumination, and lying behavior in dairy cows, comparing values from six different commercially available triaxial accelerometer systems with those from visual observations. In that study, the evaluated monitoring technologies accurately monitored the behavior of dairy cattle [50]. However, it was obvious that most of the sensors monitored only one or two behaviors, such as rumination and feeding time or only lying time. This was also noted by Paudyal [23], who concluded that further research is needed on different approaches to refine the detection algorithm and combine RT with other production and behavioral variables to obtain a system with high sensitivity and specificity of disease detection.

## 5. Conclusions

This study validated a combination of a pressure sensor and a triaxial accelerometer attached to a nose clip to measure the individual duration of rumination cycles and RT in dairy cows. The comparison of sensor data with visual observations of regurgitations showed good agreement (sensitivity: 99.1–100%, specificity: 87.8–95%) between the two methods. Future studies will focus on the automation of regurgitation detection (implementing an algorithm/use of artificial intelligence) and data cache in the microcontroller-based system to provide real-time online data. Furthermore, we will assess whether the accelerometer data also shows and can be used to calculate the chews per bolus. The advantage of this system is its continuous measurement and monitoring of individual animals and the combination of behavioral (duration of rumination cycle, RT) and physiological (respiration rate) variables recorded.

## Figures and Tables

**Table 1:** Overview of analyzed trial periods, kinds of observations, and number of regurgitation events (visually counted or sensor-based).

Year	2018	2018	2022	2022
Rumination behavior during observation period	yes	no	yes	no
Number of cows (*n*)	6	6	13	6
Method of visual observation	Video	Video	Direct	Direct
Total duration of observations (h:m:s)	03:07:10	01:00:00	06:14:47	01:00:00
Number of visually observed regurgitations (*n*)	201	0	378	0
Number of sensor-based regurgitations (*n*)	201	3	227	5
Number of visually observed regurgitations not recorded by the sensor because of a streaming gap (*n*)	0	0	149	0
Number of minutes with streaming gaps in the sensor data (*n*)	0	0	0	19
Visually observed regurgitations not visible in the recorded sensor-based data (*n*)	0	0	2	0

**Table 2:** Classification performance (in %) regarding the comparison of sensor-based regurgitation events with visually observed regurgitation events.

Year and data basis	2018	2022 (raw data)	2022 (after correction of sensor gaps)
Sensitivity (*Se*, true positive rate)	100.0	60.1	99.1
Specificity (*Sp*, true negative rate)	95.0	91.7	87.8
Positive predicted value (*PPV*)	98.5	97.8	97.8
Negative predicted value (*NPV*)	100.0	26.7	94.7

## Data Availability

The data presented in this study are available upon request from the corresponding author.
